# A genetic modifier suggests that endurance exercise exacerbates Huntington's disease

**DOI:** 10.1093/hmg/ddy077

**Published:** 2018-03-02

**Authors:** Silvia Corrochano, Gonzalo Blanco, Debbie Williams, Jessica Wettstein, Michelle Simon, Saumya Kumar, Lee Moir, Thomas Agnew, Michelle Stewart, Allison Landman, Vassilios N Kotiadis, Michael R Duchen, Henning Wackerhage, David C Rubinsztein, Steve D M Brown, Abraham Acevedo-Arozena

**Affiliations:** 1 Mammalian Genetics Unit, Harwell Institute, Medical Research Council, Oxfordshire, UK; 2 Department of Biology, University of York, York, UK; 3 Institute of Medical Sciences, University of Aberdeen, Scotland, UK; 4 Department of Cell and Developmental Biology, University College London (UCL), London, UK; 5 Department of Sport and Health Sciences, Technical University of Munich (TUM), Exercise Biology, Munich, Germany; 6 Department of Medical Genetics, Cambridge Institute for Medical Research, University of Cambridge, UK; 7 UK Dementia Research Institute, University of Cambridge, Cambridge, UK; 8 Unidad de Investigación, Hospital Universitario de Canarias, Fundación Canaria de Investigación Sanitaria e Instituto de Tecnologías Biomédicas, La Laguna, Spain

## Abstract

Polyglutamine expansions in the huntingtin gene cause Huntington’s disease (HD). Huntingtin is ubiquitously expressed, leading to pathological alterations also in peripheral organs. Variations in the length of the polyglutamine tract explain up to 70% of the age-at-onset variance, with the rest of the variance attributed to genetic and environmental modifiers. To identify novel disease modifiers, we performed an unbiased mutagenesis screen on an HD mouse model, identifying a mutation in the skeletal muscle voltage-gated sodium channel (*Scn4a*, termed ‘draggen’ mutation) as a novel disease enhancer. Double mutant mice (HD; *Scn4a^Dgn/+^*) had decreased survival, weight loss and muscle atrophy. Expression patterns show that the main tissue affected is skeletal muscle. Intriguingly, muscles from HD; *Scn4a^Dgn/+^* mice showed adaptive changes similar to those found in endurance exercise, including AMPK activation, fibre type switching and upregulation of mitochondrial biogenesis. Therefore, we evaluated the effects of endurance training on HD mice. Crucially, this training regime also led to detrimental effects on HD mice. Overall, these results reveal a novel role for skeletal muscle in modulating systemic HD pathogenesis, suggesting that some forms of physical exercise could be deleterious in neurodegeneration.

## Introduction

Huntington’s disease (HD) is an autosomal dominant neurodegenerative disorder caused by pathogenic poly-glutamine expansions in the huntingtin gene (*HTT*) (1). Overlapping pathological mechanisms operate in HD pathogenesis, such as altered calcium metabolism, transcriptional impairment, altered autophagy or mitochondrial dysfunction, amongst others. Huntingtin is ubiquitously expressed (2). Consequently, although primarily a neurodegenerative disorder, HD also causes pathological changes in many peripheral tissues and organs including skeletal muscle (3, 4). Metabolic abnormalities in HD patients and mouse models are well established, particularly a hypermetabolic state accompanied by weight loss despite normal food intake, although their causes are not entirely clear (5,6).

Together with the brain, skeletal muscle is the main consumer of glucose, especially during exercise. Thus, changes in skeletal muscle homeostasis, including those induced by physical exercise or by pathological abnormalities, can in turn affect whole body metabolism, ultimately affecting the brain. In this sense, physical exercise has been tested as a possible treatment for HD (7). A recent clinical trial showed that moderate exercise was not detrimental to motor functions in HD patients (8). Studies in different HD mouse models have shown potential beneficial effects of moderate exercise, especially observed in hippocampal neurogenesis (9,10). However, there are also several reports evidencing detrimental effects of exercise regimes in HD mice, as well as inconclusive studies in HD patients (11).

To date, many HD modifier genes have been described (12), offering potential novel therapeutic targets for further study. Here, we conducted a screen to search for novel HD modifier genes using the mouse as a complex model system. We identified the skeletal muscle voltage-gated sodium channel (*Scn4a*) as a novel modifier gene exacerbating the overall HD disease phenotype. The interaction of *Scn4a* and HD mutations leads to higher energy demands that trigger adaptation changes in skeletal muscles. These adaptations are also observed after endurance training regimes, allowing us to show that physical exercise regimes leading to higher energy demands are detrimental in HD mice.

## Results

### A genetic screen in mice identifies the skeletal muscle sodium-gated voltage channel (Nav1.4, *Scn4a*) as a Huntington’s disease modifier gene

**Figure 1.**
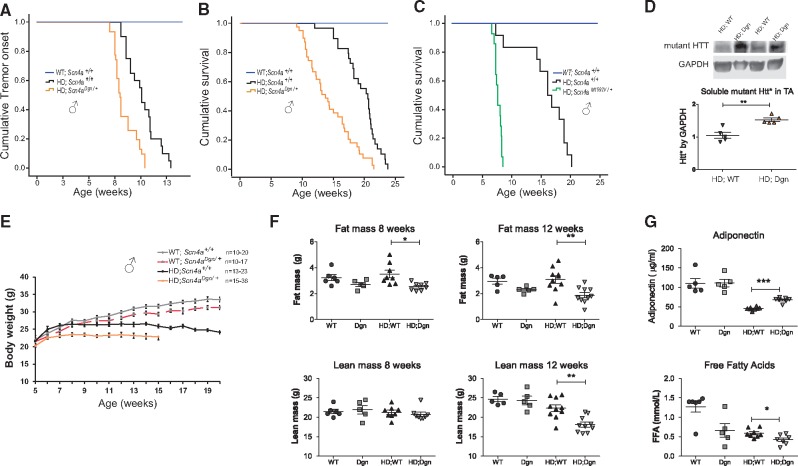
*Scn4a* mutations exacerbate HD leading to further metabolic alterations. (**A**) Cumulative tremor onset events from 7 weeks of age (HD; *Scn4a^+/+^* (*n* = 20) average onset: 10.1 weeks; HD; *Scn4a^Dgn/+^* (*n* = 31) average onset: 8.4 weeks. *P < *0.001). (**B**) Draggen mutation decrease survival on HD males (HD; *Scn4a^+/+^* (*n* = 28) average survival: 19.4 weeks; HD; *Scn4a^Dgn/+^* (*n* = 39) average survival: 14.3 weeks. *P < 0.001*. (**C**) The M1592V *Scn4a* mutation decreases survival in HD male mice (HD; *Scn4a^+/+^* (*n* = 12) average survival: 15.8 weeks; HD; *Scn4a^M1592V/+^* (*n* = 14) average survival: 7.7 weeks. *P < *0.001). (**D**) Representative immunoblot and analysis of levels of soluble mutant huntingtin from TA muscle corrected by GAPDH (*n* = 5, *P = *0.0018). (**E**) Body weights from 5 weeks of age until disease end-point. At least 13 male mice per genotype and time-point, (*P *<* *0.05 from 5 weeks onwards comparing HD; *Scn4a^Dgn/+^* to any other group). (**F**) Whole body fat and lean mass in male mice at 8 (*n* = 5 nonHD and *n* = 8 HD groups) and 12 weeks of age (*n* = 5 nonHD groups and *n* = 10 HD groups). (**G**) Free-fed circulating levels of adiponectin and free-fatty acids in plasma of male mice at 12 weeks of age (*n* = 5 nonHD and *n* = 8 HD groups). WT= WT; *Scn4a^+/+^*, Dgn= WT; *Scn4a^Dgn/+^*, HD; WT= HD; *Scn4a^+/+^* and HD; Dgn= HD; *Scn4a^Dgn/+^*. *P < 0.05, **P < 0.01, ***P< 0.001.

We conducted an unbiased forward dominant genetic screen in mice that enables the identification of novel genes involved in the systemic alterations that occur through disease progression at the whole organismal level. Briefly, BALB/cAnN males were mutagenized with *N*-ethyl-*N*-nitrosourea (ENU), causing germline-transmitted random point mutations that were crossed to congenic C57BL/6J HD transgenic females (N171–82Q) generating mutant F1 HD mice that were then subjected to phenotypic screening (Supplementary Material, Fig. S1A). After identifying individual HD phenodeviants through behavioural phenotyping, we backcrossed them onto C57BL/6J to assess their progeny for inheritance of the selected modifier effects. From the modifier screen, we assessed around 350 HD F1 mice, of which 7% were selected as potential individual phenodeviants. Of the lines subjected to inheritance testing, 4 showed confirmed inheritance. Here we focus on one of the enhancer lines, that we called draggen, carrying a mutation in the *Scn4a* gene. Interestingly, as well as enhancing the overall HD phenotype, the mutation caused a new trait, intermittent paralytic attacks, appearing also independently of the HD transgene. In previous work, we segregated the draggen mutation from the HD transgene, cloned it and characterized it as a model of *SCN4A* channelopathies, leading to non-dystrophic myotonia and intermittent attacks of hind-limb immobility followed by total recovery (13). Here we focus on the effects of *Scn4a* mutations as HD modifiers. In HD; *Scn4a^Dgn/+^* mice, the draggen mutation exacerbated the overall HD phenotype, including accelerating the age at onset of tremors in HD males as well as dramatically reducing survival (defined as the age at which a set of humane endpoints are reached) (Fig. 1A and B). Sexual dimorphism was observed in the HD modifier effect (Supplementary Material, Fig. S1B). Due to the incomplete penetrance in females, we focused on males for the rest of the study. To confirm that *Scn4a* mutations can indeed modify the HD phenotype, we crossed HD transgenic mice to an independently generated *Scn4a* allele (*Scn4a^M1592V/+^*) (14). HD mice carrying the *Scn4a* M1592V mutation (*HD; Scn4a^M1592V/+^*) enhanced HD even more severely than the draggen mutation in both males and females (Fig. 1C and Supplementary Material, Fig. S1C). Thus, *Scn4a* mutations modify the overall HD phenotype in mice.

As expected, *Scn4a* mRNA levels measured via qPCR are primarily expressed in skeletal muscle with marginal expression in the heart and practically no expression in brain; this expression pattern is not altered by the draggen mutation (Supplementary Material, Fig. S2A). We confirmed these results by analysing B-galactosidase staining on a *Scn4a* null allele carrying a LacZ reporter (Supplementary Material, Fig. S2B). Moreover, the mutant huntingtin transgene in N171–82Q mice is expressed in skeletal muscle at about half of the expression levels seen in brain (Supplementary Material, Fig. S2C). Thus, skeletal muscle is the only tissue showing high levels of expression for both genes. Due to the unique expression pattern of *Scn4a*, the study of its HD modifier effect allowed us to identify changes specifically in skeletal muscle that could be detrimental for systemic HD pathogenesis. To evaluate if the genetic interaction between the mutated forms of huntingtin and *Scn4a* could enhance HD pathology in the brain, we measured soluble mutant huntingtin and huntingtin intranuclear inclusions at piriform cortex and cerebellum (Supplementary Material, Fig. S2D and E), finding no differences between genotypes. In contrast, soluble mutant huntingtin levels were increased in HD; *Scn4a^Dgn/+^*skeletal muscles when compared to HD; *Scn4a^+/+^* littermates (Fig. 1D).

One of the most important features observed in HD patients and mouse models is severe weight loss, which is partially explained by a hypermetabolic state (15) and progressive skeletal muscle atrophy. We evaluated whether accelerated muscle wasting and cachexia could be a possible cause of the early death observed in HD*; Scn4a^Dgn/+^*mice. We observed that HD*; Scn4a^Dgn/+^*male mice suffered from further progressive weight loss when compared to draggen or HD males alone (Fig. 1E). This is reflected in a tendency towards increasing *in vivo* whole body energy expenditure of HD; *Scn4a^Dgn/+^*when compared to HD; *Scn4a^+/+^* littermates at 8 weeks of age, with no major changes in food intake at this age (Supplementary Material, Fig. S3A). The weight differences between HD; *Scn4a^+/+^* and HD; *Scn4a^Dgn/+^*males were mainly explained by a reduction in fat mass progressively until disease end-stage (Fig. 1F). Interestingly, at disease end-stage for double mutants (12 weeks of age), there is further loss of lean mass. At this end-stage, in double mutant mice, energy expenditure is still high compared to control wild-type mice (although similar to control HD; *Scn4a^+/+^* mice), also showing a tendency towards a lower food consumption (Supplementary Material, Fig. S3B). This decrease in fat mass in HD; *Scn4a^Dgn/+^* is consistent with changes in adiponectin levels and reduction of free fatty acid levels in plasma of HD; *Scn4a^Dgn/+^* male mice (Fig. 1G).

All these data together suggest that the genetic interaction between *Scn4a* and HD mutations further increases the already elevated energy turnover of HD mice, potentially depleting the body of different energy sources that may accelerate the disease in double mutant mice.

### *Scn4a* mutations lead to mitochondrial enlargement and metabolic adaptations in HD skeletal muscle

**Figure 2.**
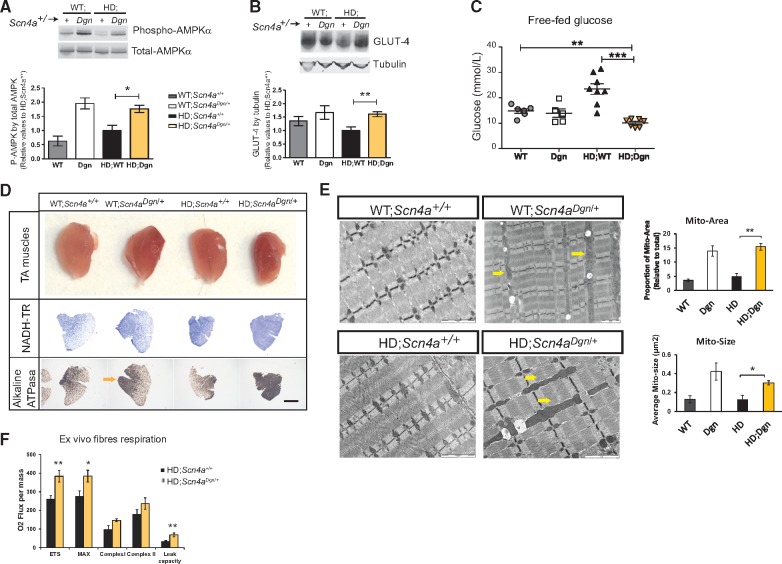
HD muscles carrying *Scn4a* mutation showed adaptive changes to higher energy demands. All analysis from male TA muscle at 12 weeks of age, unless stated otherwise. (**A** and **B**) Immunoblot of AMPKα activation levels measured via the ratio of Thr321 phosphorylated AMPK versus total AMPKα (A), and glucose transporter (GLUT-4) protein levels corrected by tubulin (B), (*n* = 3 nonHD; *n* = 4 HD groups). (**C**) Free-fed circulating levels of glucose. (**D**) Representative images of whole muscle (first row) and sections. Second and third panel rows show comparable TA transversal sections stained with NADH-TR and Alkaline ATPasa activity, showing oxidative fibre grouping in HD; *Scn4a^Dgn/+^* (yellow arrow). (**E**) Representative TEM muscle micrographs. Arrows point to the appearance of enlarged mitochondria. Scale bar represents 2 µm. Quantification of the average mitochondria area and size in TEM. Each bar represents the average of *n* = 3–5 mice per group and at least five comparable TEM per mouse. (**F**) Analysis of mitochondrial respiration (Oxygen Consumption Rate (OCR)) *ex vivo* from permeabilized fibre bundles of EDL muscle using high resolution respirometry (*n* = 5). Graph bars represent mean ± SEM and values are relative to the average HD; *Scn4a^+/+^* group value. *P< 0.05, **P < 0.01, ***P < 0.001.

We have previously observed that draggen mice when not in the context of the HD transgene (WT; *Scn4a^Dgn/+^*) have increased muscle activity (myotonia and intermittent paralytic attacks) which activated adaptive non-dystrophic changes, switching skeletal muscle fibres towards more oxidative types (13). These changes are physiological in a plastic tissue like skeletal muscle, but could be detrimental in a disease context. Thus, we first examined if these adaptive responses were also present in HD; *Scn4a^Dgn/+^* mice, as they could be contributing towards the accelerated cachexic state in double mutants. First, we focussed on AMPK activation, the master energy sensor that is activated via phosphorylation when there is a deficit in energy supply. Interestingly, AMPK is activated to similar levels in skeletal muscle of HD; *Scn4a^Dgn/+^* and WT; *Scn4a^Dgn/+^*mice, an activation likely driven by the draggen mutation alone (Fig. 2A). Despite similar AMPK activation levels in skeletal muscle between WT controls (WT; *Scn4a^+/+^*) and HD transgenic mice (HD; *Scn4a^+/+^*), the levels of the AMPK-controlled glucose transporter GLUT-4 were downregulated in skeletal muscle in HD mice when compared to non-transgenic controls (Fig. 2B), providing a potential explanation for the higher free-fed glucose levels in serum of HD mice (Fig. 2C). Interestingly, the increase in AMPK activation in HD; *Scn4a^Dgn/+^* mice leads to a normalization of GLUT-4 in skeletal muscle to levels similar to WT mice (WT; *Scn4a^+/+^*), but still higher than HD controls (HD; *Scn4a^+/+^*). As a consequence, glucose levels are significantly reduced in HD; *Scn4a^Dgn/+^* when compared to any other genotype (Fig. 2C). We validated these results by a glucose tolerance test (Supplementary Material, Fig. S3C), showing that HD; *Scn4a^Dgn/+^*are more efficient in their glucose uptake than HD controls. Overall, these results provide a potential mechanistic explanation for the reduced glucose levels observed in HD; *Scn4a^Dgn/+^*mice.

Another adaptive consequence of the activation of AMPK in response to energy deprivation is the triggering of a mitochondrial biogenesis programme, leading to a shift of muscle fibres towards more oxidative types (16). Indeed, we found an almost complete muscle fibre switching towards more oxidative types within the tibialis anterior (TA) muscle of draggen mice (in WT; *Scn4a^Dgn/+^* mice and to a greater extent in HD; *Scn4a^Dgn/+^*) (Fig. 2D and Supplementary Material, Fig. S4A and B). At the electron microscopy level, enlarged mitochondria appeared in skeletal muscle from HD; *Scn4a^Dgn/+^* mice at 12 weeks of age (Fig. 2E). This increased mitochondrial mass in HD; *Scn4a^Dgn/+^* mice was confirmed by mitochondrial DNA analysis and by increased levels of mitochondrial components of the OXPHOS respiratory chain subunits (Supplementary Material, Fig. S4C and D). It has been previously shown that mutant huntingtin interferes and disrupts the normal functioning of mitochondria at several levels (17–19). To determine whether those enlarged mitochondria were functional, we measured respiration from the dissociated muscle fibres from mice *ex vivo.* There was an increase in the overall Oxygen Consumption Rate (OCR), including a significant increase in the maximal uncoupled respiration rate of HD; *Scn4a^Dgn/+^*compared to HD; *Scn4a^+/+^*muscle bundles (Fig. 2F), which as a consequence is associated with an increased production of reactive oxygen species (ROS).

Overall, all these observations suggest that HD skeletal muscle is still capable of adapting to higher energy demands (such as the one posed by the increased activity due to *Scn4a* mutations), activating AMPK, enhancing mitochondrial function and increasing different fuel consumptions. Despite these adaptations, there is a dramatic acceleration of disease in HD; *Scn4a^Dgn/+^*mice, suggesting a detrimental effect for HD pathogenesis.

## Endurance exercise training is detrimental for HD in mice

**Figure 3.**
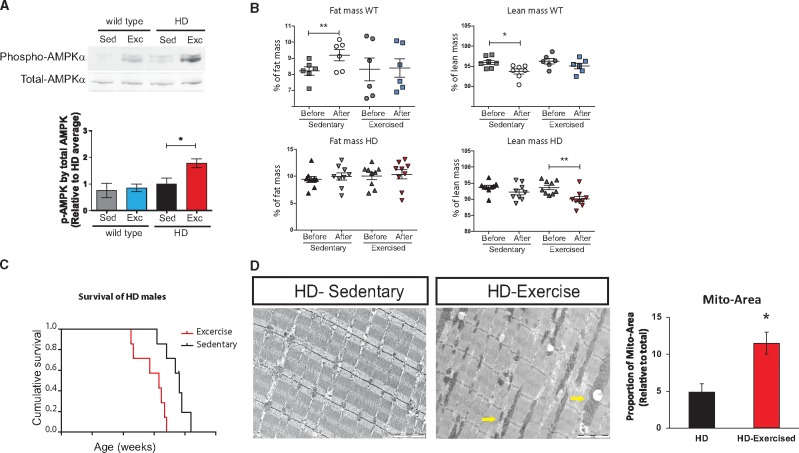
Endurance exercise is detrimental for HD. (**A**) Immunoblots for AMPKα activation in TA muscles (*n* = 4 in WT, *n* = 6 in HD groups) after 6 weeks of sedentary or exercise (**B**) Whole body fat and lean mass in male WT (*n* = 5) and HD (*n* = 7) mice before (around 7–8 weeks of age) and after 6 weeks sedentary or exercise regime. (**C**) Graph showing decrease survival in HD mice with endurance exercise (*n* = 7; *P* = 0.003). (**D**) TEM images from TA muscles of HD mice in the sedentary and exercise group (yellow arrow pointed at mitochondria). Quantification of the average mitochondria total area in TEM. Each bar represents the average of *n* = 2–4 mice per group in at least three comparable micrographs per mouse including 40–60 mitochondria per micrograph. Graph bars represent mean ± SEM. Statistical evaluation between HD groups by two-tailed *t-*Student with Welch’s correction. *P < 0.05, **P < 0.01.

The effect of *Scn4a* mutations in skeletal muscle partially resembles the adaptations induced by endurance training exercise, leading to non-dystrophic changes mediated by constitutive AMPK activation (20,21). It is very unlikely that a Huntington's patient would also carry a *SCN4A* mutation. However, HD patients could carry other genetic variations affecting their energy balance and/or skeletal muscle activity, as well as variations modulating the response to environmental stimuli such as physical exercise. In this context, the effects of physical exercise in HD pathogenesis are still controversial, but are currently considered as a potential intervention that could be beneficial for HD (7) and other neurodegenerative disorders. However, according to our results, adaptations induced by increasing energy demands in skeletal muscle, including endurance exercise, might be detrimental for HD pathogenesis. Thus, to test this hypothesis, we established an endurance training routine for HD and WT littermate male mice, starting at 7 weeks of age and lasting for 6 weeks. First, we showed that the regime led to AMPK activation in skeletal muscle of HD mice (Fig. 3A). We studied body composition before and after the endurance exercise regime, showing that exercised HD mice lose lean mass faster than sedentary HD controls by 12 weeks of age (Fig. 3B). Crucially, HD mice undergoing endurance training reached the humane endpoints significantly earlier than their sedentary HD littermates (Fig. 3C). Remarkably, and concordantly with the findings in HD; *Scn4a^Dgn/+^* muscles, enlarged mitochondria were also present in exercised HD mice, but not in their sedentary littermates at 13 weeks of age (Fig. 3D). Thus, at least in the mouse, endurance training leading to constitutive activation of AMPK in skeletal muscle is detrimental for HD pathogenesis.

## Discussion

Through an unbiased genetic screen in mice we have identified *Scn4a* as a novel HD modifier. As *Scn4a* is primarily expressed in skeletal muscle, analysis of the modifier effect highlighted a novel critical role of the periphery in modulating systemic HD pathogenesis. The phenotypic modification occurs through a mechanism mediated by AMPK activation inducing skeletal muscle adaptations leading to high-energy demands. We propose that this energetic imbalance produced by skeletal muscle adaptations ultimately exacerbates systemic HD progression. Thus, to test this hypothesis independently of *Scn4a* mutations, we subjected HD mice to an endurance training regime that phenocopies the adaptive changes present in skeletal muscle of HD; *Scn4a^Dgn/+^* mice. As we anticipated, endurance exercise was detrimental for HD mice. Thus, the critical linking point between the two sets of experiments is that they both highlight a critical role for the periphery in modulating systemic HD pathogenesis. Overall, using two independent approaches in mice, we showed that skeletal muscle adaptations mediated by constitutive AMPK activation are detrimental for systemic HD pathogenesis.

Mitochondrial impairment and energy balance dysregulation have been previously associated with HD pathogenesis (15, 22,23). Thus, interventions aimed at increasing energy production or mitochondrial biogenesis either by genetic means, physical exercise or drugs, have been proposed and tested as potential treatments for HD and other neurodegenerative disorders. Moreover, a recent clinical trial showed that moderate exercise stabilized motor functions in HD patients, although bigger study sizes are needed to assess other effects (8). However, there are several reports evidencing detrimental effects of exercise regimes in HD mice (24) as well as inconclusive studies in HD patients (11). Furthermore, activation of AMPK by injection of the AMPK-activating drug AICAR increased neuronal death and decreased lifespan in HD mice (25). Our data here provides a potential explanation for the inconsistencies on the effects of physical exercise in HD pathogenesis, as some regimes but not others may lead to adaptive changes in skeletal muscle. In light of our data, the potential benefits of augmenting mitochondrial function in HD may be enhanced when accompanied by increasing fuel supply.

Overall, our findings suggest caution to interventions aimed at HD patients that may lead to higher energy demands, including stringent physical exercise or drugs aimed at increasing mitochondrial function.

## Materials and Methods

### Mice

Mice were kept under a controlled 12-h light cycle and had free access to water and were fed ad libitum on a commercial diet (SDS). Draggen mice were identified through a dominant modifier ENU screen (26) injecting BALB/c male mice with ENU and crossing them with (N171–82Q) HD hemizygous carrier females on a C57BL/6J background. All G1 mice produced were genotyped for the HD transgene and were subject to a behavioural screening pipeline including: general dysmorphology and SHIRPA analysis, weight and grip-strength measurements. Identified individual G1 pheno-deviant mice that showed novel phenotypes or modulate the HD disease onset of progression of the parental N171–82Q HD line on a C57BL/6J-BALB/c hybrid background were selected for further analysis and backcrossed onto C57BL/6J to assess inheritance. After inheritance was established, a positional cloning approach was followed using a panel of single nucleotide polymorphisms (SNPs) polymorphic between the two parental strains.

The draggen mutation was first identified in the context of the HD transgene through the ENU modifier screen. After backcrossing to C57BL/6J, the draggen mutation was segregated from the HD transgene. The cloning of the draggen mutation and the characterization of the draggen mice when not in the context of the HD mutation has already been published, using the characteristic intermittent attacks of hind-limb immobility as the initial phenotypic selected trait. The draggen official allele name is: *Scn4a^m1Aaa^*. Mice homozygous for the draggen mutation are not viable, therefore all the work performed here is on heterozygous draggen carriers.

Draggen mice used here are at least N3 (three generations of backcross onto C57BL/6J); the colony is currently backcrossed for >10 generations onto C57BL/6J (>N10). All mice produced for this study were obtained by intercrossing both mutations in heterozygosis, producing all four possible genotypes from the same cross (WT; *Scn4a^+/+^*, WT; *Scn4a^Dgn/+^*, HD; *Scn4a^+/+^* and HD; *Scn4a^Dgn/+^*), allowing to use littermate controls. Experiments were performed blind to genotype and lifespan was defined by mice reaching one of the following humane end-points: loss of 20% of maximum body weight, the presence of hunched posture and piloerection or the appearance of hind-limb paralytic attacks lasting longer than 1 min with no full recovery.

*Scn4a^M1592V/+^* (FVB.129S4(B6)-*Scn4a^tm1.1Ljh^*/J) mice were purchased from Jackson laboratories (USA) on a congenic FVB background and generated the four desired genotypes by crossing WT; *Scn4a^M1592V/+^* to HD; *Scn4a^+/+^* mice. *Scn4a^M1592V/+^* mice were maintained in a hybrid C57BL/6J-FVB background. All genotyping was performed by pyrosequencing or light scanner (For primer list used, see Supplementary Material, Table S1).

*Scn4a* deficient mice (*Scn4a^tm2b(KOMP)Wtsi^*) were produced at MRC Harwell through the IMPC programme and are distributed through the European Mouse Mutant Archive (www.infrafrontier.eu). Cre expression deleted the critical exon, producing a lacZ tagged null allele.

### Phenotyping tests

The number of animals was chosen based on previous studies. For *in vivo* behavioural tests we required a minimum of seven mice per sex and genotype. Modified SHIRPA methods were performed as described previously (27,28). Tremors were qualitatively assessed by observation in a viewing jar and recorded as having no tremors (0), mild tremors (1), moderate tremors (2) or severe tremors (3). Mice were subject to modified SHIRPA analysis from 7 weeks of age until they reached their humane endpoint. Paralytic attacks were evaluated weekly as previously described (13).

Metabolic rate was measured at 8 and 12 weeks of age, with measurements performed for 22 h during the light and dark period, using indirect calorimetry TSE systems to determine Energy Expenditure (E.E.). Fat and lean mass in the body were determined by EchoMRI (EchoMRI-100™) scan system. For intraperitoneal glucose tolerance test (IPGTT), mice were fasted overnight (16 h) to establish a baseline glucose level ‘T_0_’ (time zero). A blood sample was collected from the tail vein after administration of local anesthetic. Mice were then injected intraperitoneally with 2 g of glucose per kg body weight (20% glucose in 0.9% NaCl). Blood samples were taken at 15, 30, 60 and 120 min after injection and glucose was measured using Alphatrak2 glucose analyser (Abbott Laboratories, USA).

### Endurance exercise challenge

Wild type and HD N171–82Q male mice littermates on a congenic C57BL/6J background had 30 min of forced exercise in the rotarod at constant speed (15 rpm), 5 days a week, plus a running wheel in their home cages, allowing for additional voluntary exercise. The routine started from pre-symptomatic (around 7 weeks of age) until they reached the humane end points in the case of HD mice (for survival analysis), or until 12 weeks of age for nonHD mice and for HD mice used for molecular analysis. Wild type and HD littermates were used for the sedentary group controls. Fat and lean mass in the body were determined by EchoMRI before (at the beginning of the experiment) and after 5 weeks of training.

### Blood parameters

Terminal blood samples were collected from mice aged 12 weeks (*n* = 5–7 per group). Mice were fasted 4 h prior to blood collection. Blood samples were collected under terminal isofluorane inhalation anaesthesia by retro-orbital puncture into paediatric lithium heparin coated tubes. Lithium heparin samples were kept on wet ice until being centrifuged for 10 min at 8000*g* at 4°C. The resulting plasma was analysed on board a Beckman Coulter AU680 clinical chemistry analyser using reagents and settings recommended by the manufacturer. Plasma mouse adiponectin was measured using an ELISA kit from Life Technology.

### Respiration analysis in muscles *ex vivo*

Respiration measurements were made from muscle bundles *ex vivo* using a high-resolution respirometry system Oxygraph-2k (Oroboros Instruments, Bioblast) which allows for the analysis of mitochondrial respiratory chain activity from mouse muscle fibers. We compared oxygen consumption rates (OCR) of extensor digitorum longus (EDL) muscle from male mice aged 12 weeks (HD; *Scn4a^+/+^ n* = 6, and HD; *Scn4a^Dgn/+^ n* = 5). Briefly, dissected EDL muscles were placed in ice-cold BIOPS solution (10 mm Ca-EGTA buffer, 0.1 µm free calcium, 20 mm imidazole, 20 mm taurine, 50 mm K-MES, 0.5 mm DTT, 6.56 mm MgCl2, 5.77 mm ATP, 15 mm phosphocreatine, pH 7.1) and were separated lengthways to obtain bundles of fibres. Three bundles per EDL were permeabilized with saponin for 30 min in ice-cold BIOPS, prior to washing and transferring to the mitochondrial medium for OCR measurements in the Oroboros chambers according to the manufacturer’s protocol. A specially designed substrate/inhibitor titration approach allowed the step-by-step analysis of the activity of the respiratory chain (RC) as a whole as well as the relative contribution to total activity of RC complexes I and II, the two entry points for electrons. Additions of substrates and inhibitors were in the following order and final concentration: Malate 2 mm, Glutamate 10 mm, ADP 2.5 mm, Cytochrome *c* 10 µm, Succinate 10 mm, titrations of 0.25 µm with the protonophore uncoupler Carbonyl cyanide 4-(trifluoromethoxy)phenylhydrazone (FCCP) until maximal uncoupled respiration was reached, Rotenone 0.5 µm, and finally Antimycin A 2.5 µm. State 2 respiration was obtained following additions of Malate and Glutamate, State 3 after ADP, Maximal uncoupled after FCCP. The relative proportion of total respiratory chain activity dependant on either of the two entry points of the RC (i.e. Complex I and II) were measured as the Rotenone sensitive component of Maximal Uncoupled respiration for complex I and the remaining activity as that for Complex II. After the experiment, muscle bundles were recovered, homogenized and total protein concentration was measured in order to correct the OCRs by the total amount of protein per chamber. For the acquisition and analysis of data the manufacturers DatLab software was used (Oroboros Instruments, Austria).

### Histology

Tibialis anterior (TA) from male mice for the four genotypes (*n* = 3 per group) were dissected at 12 week of age, weighed and snap frozen and 10 µm sections were cut in a cryostat. TA sections were stained for NADH-tetrazolium reductase and ATPasa.

To detect mutant human huntingtin intranuclear inclusions in the brain, we conducted the same protocol as previously described (29). Mice were transcardially perfused with 4% paraformaldehyde (PFA), brain removed and placed in PFA for another 4–6 h. After 30% sucrose protection, brains were crypreserved at −80° until used. Free-floating cryosections at 30 µm were immunostained against mutant huntingtin using mouse anti- human polyQ (MAB5374 Millipore, 1:500) overnight. The seconday antibody was Alexa 488 (Life Technology) and nuclear DAPI counterstaining (Prolong, Life technology). Confocal images were taking using Zeiss LSM 700 microscope at 60× amplification of the granular layers in the cerebellum. Final images stack projections (typically the sum of 8 images per projection) were used to count the total of inclusions per field. A minimum of three fields per slice, over 3 or 4 slices were used to account for the total number of inclusions per mouse. Volocity software 5.4.1 (Perkin Elmer, USA) was used to analyse the total count and classified by the size of the inclusions per image. The given data is a relative proportion of each size group to the total, falling into four categories, from the smallest aggregates (0–2 µm^2^) to the biggest inclusions found (>6 µm^2^).

### Transmission electron microscopy

Mice were perfused with a mix of 2% glutaraldehyde and 2% paraformaldehyde in PBS. TA muscles were dissected and placed in 1% OsO4 on ice for 1 h followed by two 5 min washes with Phosphate buffer (100 mm NaH_2_PO4/Na_2_HPO_4_.2H_2_0). The samples were dehydrated through an ethanol series (30%, 50%, 70%, 95% and absolute) and transferred to a 1: 1 mixture of Epon/Araldite and acetone and incubated overnight with mixing. The solution was then replaced with a 1: 1 mixture of 100% Epon/Araldite and incubated for 6 h, followed by another replacement with fresh Epon/Araldite and overnight incubation with mixing. Samples were transferred to fresh Epon/Araldite in embedding molds and oriented as required. Polymerization of the Epon/Araldite mold was completed at 65°C for 48 h. 70 nm sections were produced and stained with saturated uranyl acetate and Reynolds’ lead citrate. All images were obtained with a Tecnai 12 BioTWIN made by FEI, Eindhoven. Quantification of the TEM images was done by Image J software.

### Immuno-blot analysis

Snap frozen TA muscles and forebrain from male 12 week-old male mice were homogenized in RIPA buffer (150 mm NaCl, 1% NP40, 0.5% Na deoxycholate, 0.1% SDS, 50 mm Tris pH 7.5) with phosphatase and protease inhibitor cocktails (Roche), using lysing matrix tubes D (MP Biomedicals, Germany) and a Fast-Prep-24 homogenizer at 4°C. Homogenates were centrifuged at 12 000*g* 4°C for 20 min. 40 μg of soluble fractions were resolved by SDS–PAGE (NUPAGE system, Invitrogen) and transferred to low fluorescent PVDF (PVDF-LF) membranes (Millipore) for western-blot analysis. The following primary antibodies were used: rabbit monoclonal anti-actin (A2066, Sigma, 1:3000); mouse anti-α tubulin (ab7291, abcam, 1:3000), mouse anti-GAPDH (ab8245, abcam, 1:5000); mouse anti-soluble human polyQ (MAB1537–1C2, Millipore, 1:800), rabbit anti-p-AMPKα (Thr-172, 2535–40H9) and mouse anti-total AMPKα (2793, both Cell Signalling, 1:1000); rabbit anti-GLUT-4 (CBL243, Millipore). Total protein loading and transfer was also quantified using REVERT kit (Li-Cor Biosciences). Protein was visualized using anti-mouse or anti-rabbit secondary antibodies IRDye^®^ (Li-Cor Biosciences) at 1: 10 000 dilutions and quantified using the infrared Odyssey imaging system (Li-Cor Biosciences).

### Mitochondria DNA analysis

Extensor Digitorum Longus fast muscles were used to quantify the amount of mitochondria DNA in all experimental groups by quantitative PCR (males, *n* = 7 per group). DNA extraction was performed using DNA-blood and tissues kit (Qiagen) following manufacturer’s instructions. The sequences of the primer used are detailed in Supplementary Material, Table S1.

### Gene expression analysis

RNA extraction was performed using RNeasy kit (Qiagen) from whole brain and Quadriceps. The quantity of the RNA was checked using a nanodrop and the quality was assessed using Bioanalyser. RINs of over 7.5 were always observed. cDNA synthesis was performed using the High Capacity cDNA RT kit (ThermoFisher Scientific) starting with 2 µg of RNA. cDNA was at a final concentration of either 20 or 50 ng per well depending upon the gene being studied. All the reactions were run in triplicate. The real-time experiments were performed on a 7900 fast machine (ThermoFisher Scientific). For the probe based experiments, TaqMan Fast Universal PCR Master was used and the reactions had a final volume of 20 µl, and Taqman assays (ThermoFisher Scientific) used as per manufactures recommendations. For the sybr experiments; fast Sybr Green mastermix from ThermoFisher Scientific was used and the reactions had a final volume of 20 µl. Primers were at a final concentration of 360 nm. Primers were designed to span exon-exon boundaries and are listed in Supplementary Material, Table S1. Fold changes were calculated using the 2-ddCt method using the 7500 Software v2.0.6 and normalized using S16 or S18 endogenous reference genes relative to HD; *Scn4a^+/+^* genotype (30).

### Statistical analysis

Error bars represented SEM. *P* values of <0.05 were considered significant. *P* values comparing two HD groups were calculated using the two-tailed Student's *t*-test (with Welch’s correction or Mann–Whitney when appropriate) and ANOVA with Tukey postHoc analysis. Log-Rank test was used for survival and age at onset analysis, and ANCOVA regression model for Energy Expenditure analysis.

### Study approval

All animal studies were carried out in accordance with UK Home Office legislation and local ethical guidelines. Mice characterization followed ARRIVE guidelines.

## Supplementary Material

Supplementary Material is available at *HMG* online.

## Supplementary Material

Supplementary Data
