# Supplementary material for: A genetic modifier suggests that endurance exercise exacerbates Huntington's disease
Source: Hum Mol Genet. 2018 Mar 2;27(10):1723–31. doi: 10.1093/hmg/ddy077 (PMC5932560; doi:10.1093/hmg/ddy077)
Supplement: Supplementary Data [file ddy077_suppl_data.zip › Supplemental Figure 2.pdf]

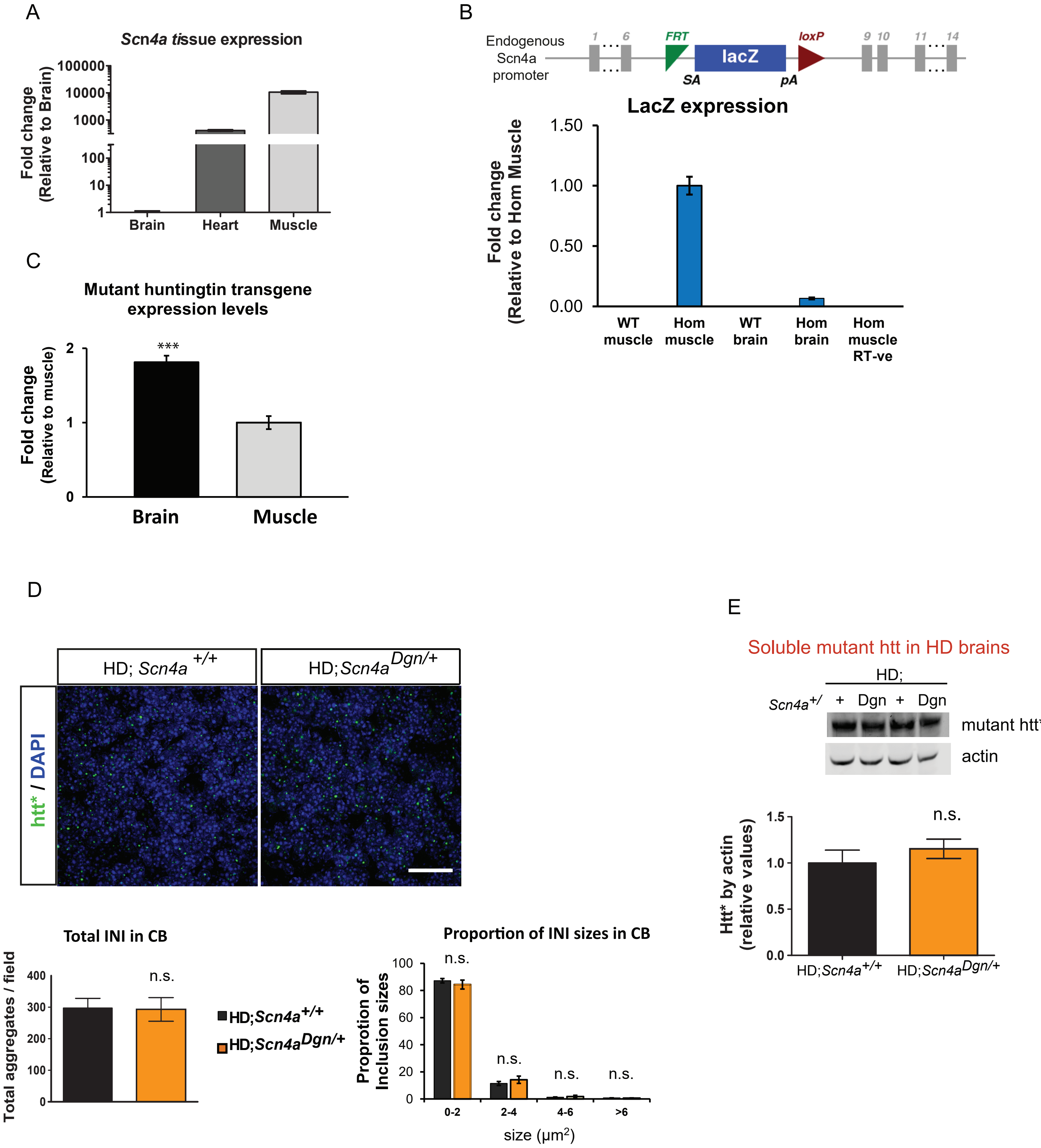

A
Scn4a tissue expression
Fold change (Relative to Brain)
100000
10000
1000
100
10
1
Brain
Heart
Muscle
B
Endogenous Scn4a promoter
1
6
FRT
lacZ
loxP
9
10
11
14
SA
pA
LacZ expression
Fold change (Relative to Hom Muscle)
1.50
1.00
0.50
0.00
WT muscle
Hom muscle
WT brain
Hom brain
Hom muscle RT-ve
C
Mutant huntingtin transgene expression levels
Fold change (Relative to muscle)
2
1
0
***
Brain
Muscle
D
HD; Scn4a +/+
HD;Scn4a Dgn/+
htt* / DAPI
Total INI in CB
Total aggregates / field
400
300
200
100
0
n.s.
HD;Scn4a+/+
HD;Scn4aDgn/+
Proportion of INI sizes in CB
Proportion of Inclusion sizes
100
80
60
40
20
0
n.s.
n.s.
n.s.
n.s.
0-2
2-4
4-6
>6
size (μm2)
E
Soluble mutant htt in HD brains
HD;
Scn4a+/
+
Dgn
+
Dgn
mutant htt
actin
Htt* by actin (relative values)
1.5
1.0
0.5
0.0
n.s.
HD;Scn4a+/+
HD;Scn4aDgn/+
