# Supplementary material for: A genetic modifier suggests that endurance exercise exacerbates Huntington's disease
Source: Hum Mol Genet. 2018 Mar 2;27(10):1723–31. doi: 10.1093/hmg/ddy077 (PMC5932560; doi:10.1093/hmg/ddy077)
Supplement: Supplementary Data [file ddy077_suppl_data.zip › Supplemental Figure 3.pdf]

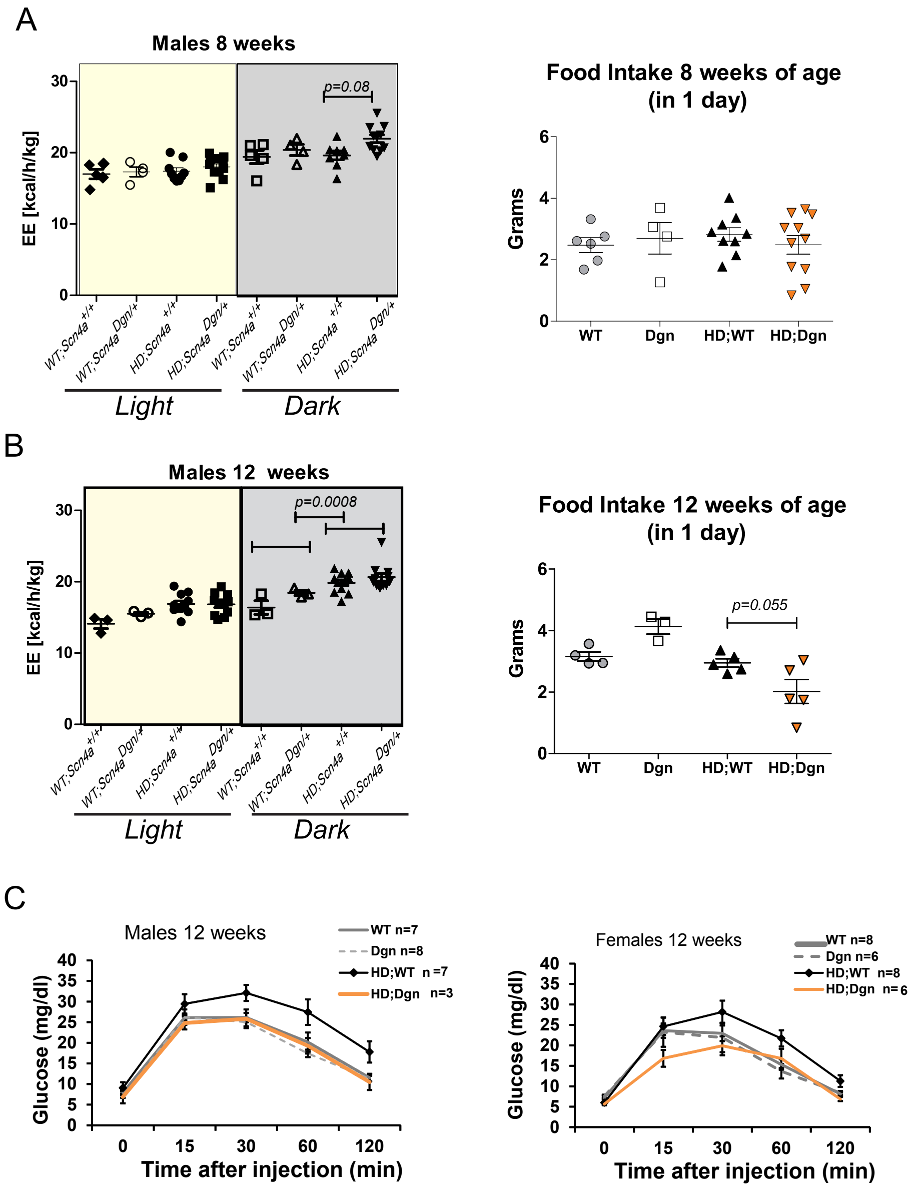
A
Males 8 weeks
p=0.08
EE [kcal/h/kg]
30
20
10
0
WT;Scn4a +/+
WT;Scn4a Dgn/+
HD;Scn4a +/+
HD;Scn4a Dgn/+
Light
Dark
Food Intake 8 weeks of age
(in 1 day)
Grams
6
4
2
0
WT
Dgn
HD;WT
HD;Dgn
B
Males 12 weeks
p=0.0008
EE [kcal/h/kg]
Light
Dark
Food Intake 12 weeks of age
(in 1 day)
p=0.055
Grams
WT
Dgn
HD;WT
HD;Dgn
C
Males 12 weeks
WT n=7
Dgn n=8
HD;WT n =7
HD;Dgn n=3
Glucose (mg/dl)
40
35
30
25
20
15
10
5
0
0
15
30
60
120
Time after injection (min)
Females 12 weeks
WT n=8
Dgn n=6
HD;WT n=8
HD;Dgn n= 6
Glucose (mg/dl)
Time after injection (min)
