# Supplementary material for: A genetic modifier suggests that endurance exercise exacerbates Huntington's disease
Source: Hum Mol Genet. 2018 Mar 2;27(10):1723–31. doi: 10.1093/hmg/ddy077 (PMC5932560; doi:10.1093/hmg/ddy077)
Supplement: Supplementary Data [file ddy077_suppl_data.zip › Supplementary data.docx]

**Supplementary Figure legends**

**Supplementary Figure 1. *Scn4a* identified in modifier screen in HD mice.**

(**A**) ENU dominant modifier screen strategy. (m/+ =ENU mutant carrier; X= crossing; arrow= following generation).

(**B**) The expression level of the mutant *Scn4a* allele in the heterozygous *Scn4a^Dgn/+^* mice is not affected by the HD transgene in the skeletal muscle, analyzed by pyrosequencing (n=4 mice per group. Unpaired *t-*Student 2-tailed, *p=*0.8).

(**C**) Female Tremor onset (HD;*Scn4a^+/+^* (n=16) average onset: 11.1 weeks; HD;*Scn4a^Dgn/+^* (n=19) average onset: 10.4 weeks. Log-rank comparison *p=0.207*), and survival (HD;*Scn4a^+/+^* (n=33) average survival: 21.9 weeks; HD;*Scn4a^Dgn/+^* (n=27) average survival: 20.1 weeks. Log-rank comparison *p=0.007*).

(**D**) Decrease survival on HD females carrying the M1592V *Scn4a* mutation. (HD;*Scn4a^+/+^* (n=13) average survival: 18.3 weeks; HD;*Scn4a^M1592V/+^* (n=11) average survival: 11.3 weeks. Log-rank *p<0.001*).

**Supplementary Figure 2. Skeletal muscles express *Scn4a* and mutant huntingtin at high levels.**

(**A**) Comparison of *Scn4a* gene expression levels in muscle, heart and brain from 12 week-old male mice by qPCR (n=12 mice from the four genotypes). Relative values are normalised to the residual detected brain expression.

(**B**) Skeletal muscle and brain from *Scn4a* KO mice from the IMPC consortium carrying a LacZ reporter allele were evaluated for *Scn4a* promoter-driven LacZ gene expression. Again, residual LacZ brain gene expression was detected. RT-ve is the negative control without retro-transcriptase enzyme.

(**C**) mRNA HD transgene expression levels quantified by qPCR in brain and muscles of HD; *Scn4a^+/+^*male mice (n=4). Unpaired *t-*Student 2-tailed with Welch’s correction *p<*0.0001.

**(D)** Analysis of mutant huntingtin inclusions in the cerebellum of HD mice. Representative confocal projection images showing mutant huntingtin aggregates (green) and DAPI nuclear staining (blue) in the granular layer of cerebellum of HD; *Scn4a^+/+^* (n= 8) and HD; *Scn4a^Dgn/+^* (n= 5) mice at 12 weeks of age. Graph bars showing the average total count of mutant huntingtin inclusions (per field at 60X amplification) in the cerebellum and an automated quantification according to aggregate size (ranging from small, up to 2 µm^2^, medium between 2 and 4 µm^2^, and large 4 to 6 µm^2^ and very big over 6 µm^2^). Scale bar represents 20 µm. Unpaired *t-*Student 2-tailed test.

(**E**) Immunoblot analysis representation of soluble mutant huntingtin in brain homogenates of HD male mice (n=4) corrected by actin loading protein control.

Graph bars represent mean ± SEM. ****p< 0.001, **p<0.01* and **p<0.05.*

**Supplementary Figure 3. Energy expenditure analysis during 24-hour period in males at 8 and 12 weeks of age.** Yellow area =averages of 12h-light measurements. Grey area= 12-hours dark measurements.

(**A**) At 8 weeks of age, there is a clear tendency of higher energy expenditure only observed in the double mutant mice, compared to their HD littermate controls (*ANCOVA* *p=*0.08). Graph representing the average food consumption in 23-hour period at 8 weeks of age (n=6-10 per group).

(**B**) Energy expenditure analysis in males at 12 weeks of age (n=3-10 mice per group). Graph representing the average food consumption in 23-hour period at 12 weeks of age (n=3-5 per group.

(**C**) Glucose tolerance test in HD male mice (HD;*Scn4a^+/+^* n=7; HD;*Scn4a^Dgn/+^*, n=3) and in females (n=6-8) at 12 weeks of age. Only 3 HD;*Scn4a^Dgn/+^* male mice could be used due to welfare reasons. (WT= WT;*Scn4a^+/+^*, Dgn= WT;*Scn4a^Dgn/+^*, HD;WT= HD;*Scn4a^+/+^* and HD;Dgn= HD;*Scn4a^Dgn/+^*. )**P< 0.05, **P< 0.01, ***P< 0.001*.

**Supplementary Figure 4. Muscle fibre type changes and mitochondria biogenesis observed in double mutant mice.**

(**A**) RNA expression levels of several myosin genes representative of the fibres types in quadriceps muscles of the four genotypes (n=5 per genotype).

(**B**) Graph showing the quantification of mitochondria DNA (mtDNA) in EDL (Extensor Digitorum Longus) muscles, using GAPDH as nuclear gene reference (n=6 nonHD; n=7 HD groups).

(**C**) Representative immunoblots showing the levels of subunits in the complexes that form the mitochondrial oxidative phosphorylation (OXPHOS) system, together with TFAM (inducing factor of mitochondrial biogenesis) (n=3 in non HD and n=4 in HD groups). Unpaired *t-*Student 2-tailed test for comparison between the two HD groups. Data are expressed as mean ± SEM. ***p<0.01* and **p<0.05.*

**Supplementary Table 1.**

|  |  |  |  |
| --- | --- | --- | --- |
|  |  |  |  |
|  |  | **(*Scn4a*-I582V) by light scanner:** |  |
|  | **Gene** | **Sequence Forward** | **Sequence Reverse** |
|  | *Scn4a*-I582V | CGTGGGTGAAGTTCAAGC | CCCACAGAGAGCACGTT |
|  |  | **Probe** |  |
|  |  | GTTGAGCACGACGCAGATGGTGAT |  |
|  |  |  |  |
|  |  | **Pyrosequencing primers for**  **Draggen mice (*Scn4a*-I582V):** |  |
|  | **Gene** | **Sequence Forward Biot** | **Sequence Reverse** |
|  | *Scn4a*-I582V | [btn]GACCTGGGCATCACCATCT | AAGTGCTCAGTCATGGGGTAGTG |
|  |  | **Sequencing primer-Reverse** |  |
|  |  | GGGTGTTGAGCACGA |  |
|  |  |  |  |
|  |  | **Pyrosequencing primers for *Scn4a*-M1592V mice:** |  |
|  | **Gene** | **Sequence Forward Biot** | **Sequence Reverse** |
|  | *Scn4a*-M1592V | [btn]CTTCCTCATCGTGGTCAA | TCTCGTAGAACATCTCAAAGTCA |
|  |  | **Sequencing primer-Reverse** |  |
|  |  | GGATGATGGCGATGTA |  |
|  |  |  |  |
|  |  | **Taqman assays for gene expression (Life Technology)** |  |
|  | **Gene** | **Taqman assay** |  |
|  | *Scn4a* | Mm00500103_m1 |  |
|  | *Rnr2* | Mm04260181_s1 |  |
|  | *Hprt1* | Mm01545399_m1 |  |
|  |  |  |  |
|  | **Gene** | **Sequence Forward** | **Sequence Reverse** |
|  | *LacZ* | CTCGCCACTTCAACATCAAC | TTATCAGCCGGAAAACCTACC |
|  |  | **Probe** |  |
|  |  | TCGCCATTTGACCACTACCATCAATCC |  |
|  |  |  |  |
|  |  | **Mitochondria DNA copy number with Sybr Green** |  |
|  | **Gene** | **Sequence Forward** | **Sequence Reverse** |
|  | *Gapdh* | CAAGGAGTAAGAAACCCTGGACC | CGAGTTGGGATAGGGCCTCT |
|  | *mtND1* | CCCATTCGCGTTATTCTT | AAGTTGATCGTAACGGAAGC |
|  |  |  |  |
|  |  | **Gene expression with Sybr Green** |  |
|  | **Gene** | **Sequence Forward** | **Sequence Reverse** |
|  | *Myh1* | GCGAATCGAGGCTCAGAACAA | GTAGTTCCGCCTTCGGTCTTG |
|  | *Myh2* | ACTTTGGCACTACGGGGAAAC | CAGCAGCATTTCGATCAGCTC |
|  | *Myh4* | CTTTGCTTACGTCAGTCAAGGT | AGCGCCTGTGAGCTTGTAAA |
|  | *Myh7* | ACTGTCAACACTAAGAGGGTCA | TTGGATGATTTGATCTTCCAGGG |
|  | *Scn4a* | TCTCCTCCTCCACCATCATC | CACCCTCCTCCATCTCAAAG |
|  | *S16* | TTCTGGGCAAGGAGCGATT | GATGGACTGTCGGATGGCA |
|  |  |  |  |
|  |  |  |  |
|  |  |  |  |
