# Supplementary figures and images for: A genetic modifier suggests that endurance exercise exacerbates Huntington's disease
Source: Hum Mol Genet. 2018 Mar 2;27(10):1723–31. doi: 10.1093/hmg/ddy077 (PMC5932560; doi:10.1093/hmg/ddy077)

A

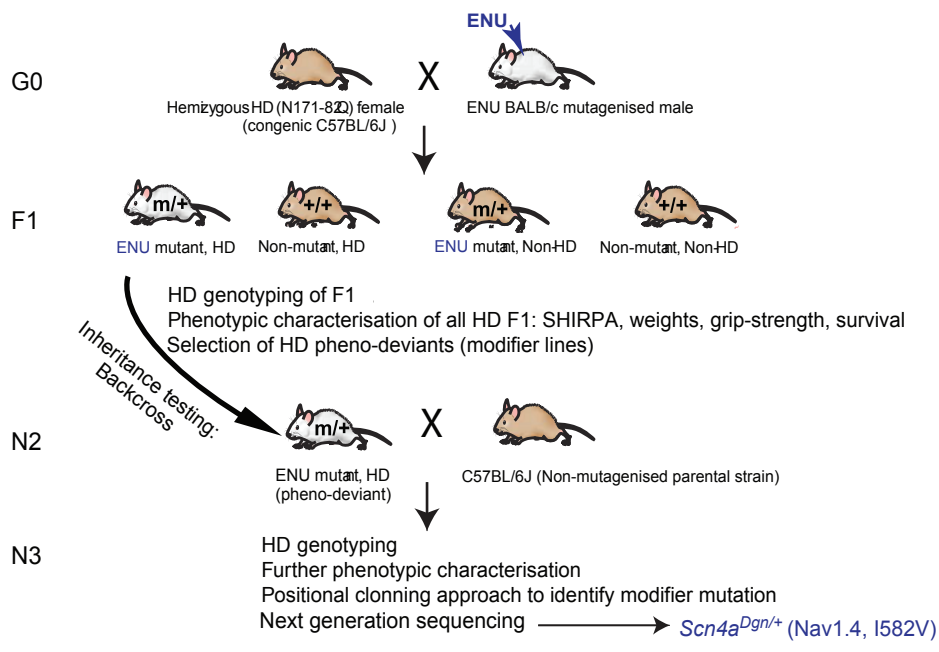

B

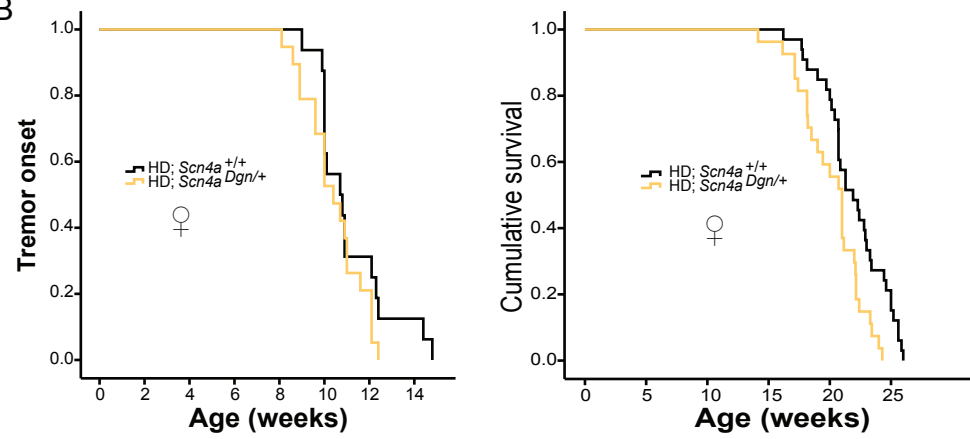

C

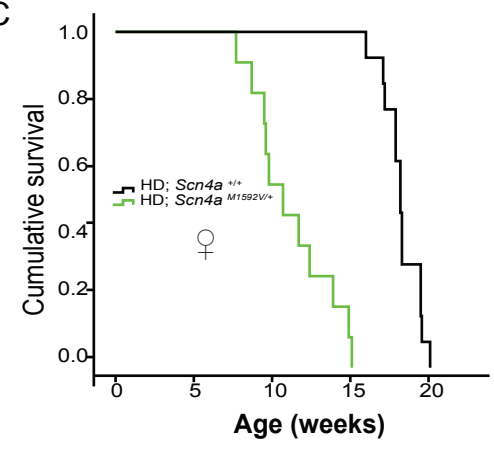

Supplement: Supplementary Data [file ddy077_suppl_data.zip › Supplemental Figure 1.pdf]

A

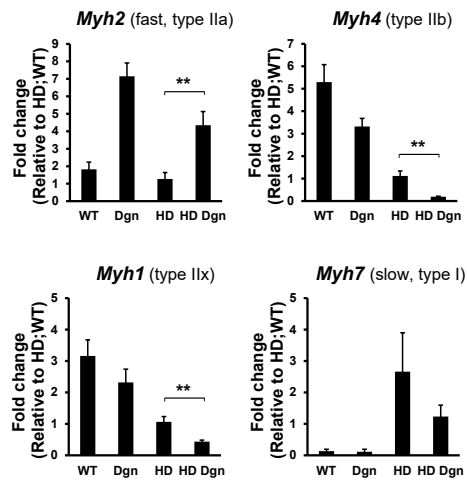

B

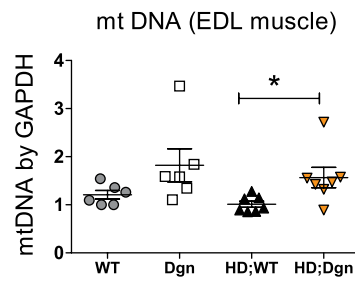

C

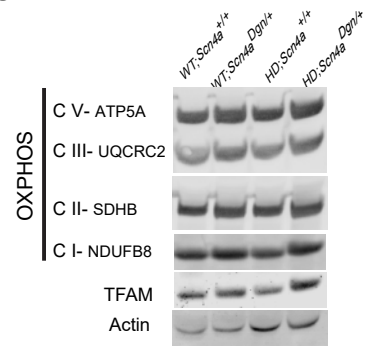

Supplement: Supplementary Data [file ddy077_suppl_data.zip › Supplemental Figure 4-Brain.pdf]
